# Supplementary material for: The interactome of intact mitochondria by cross-linking mass spectrometry provides evidence for coexisting respiratory supercomplexes
Source: Mol Cell Proteomics. 2017 Dec 8;17(2):216–32. doi: 10.1074/mcp.RA117.000470 (PMC5795388; doi:10.1074/mcp.RA117.000470)
Supplement: Supplemental Data [file supp_RA117.000470_133922_0_supp_23978_fzffwf.zip › spectra_annotation/mito_DR_spectra_annotation/416-1-4-1-2-1.pdf]

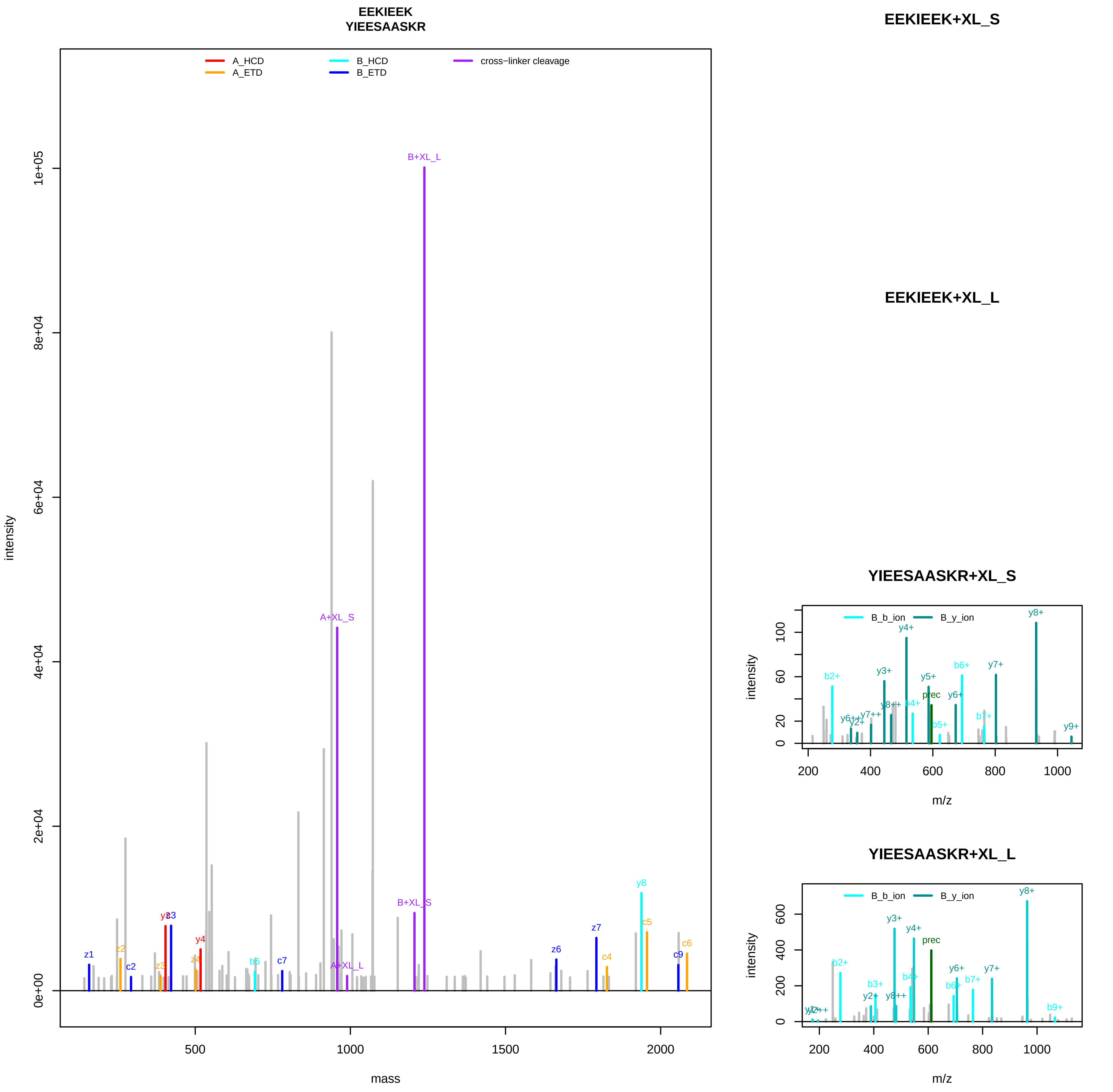
EEKIEEK
YIEESAASKR
A_HCD
A_ETD
B_HCD
B_ETD
cross−linker cleavage
B+XL_L
A+XL_S
B+XL_S
A+XL_L
z1
z2
c2
z3
y3
c3
z4
y4
b6
c7
z6
z7
c4
y8
c5
c9
c6
intensity
mass
0e+00
2e+04
4e+04
6e+04
8e+04
1e+05
500
1000
1500
2000
EEKIEEK+XL_S
EEKIEEK+XL_L
YIEESAASKR+XL_S
B_b_ion
B_y_ion
b2+
y3+
y4+
y5+
prec
b6+
y6+
y7+
y8+
y9+
b4+
b5+
b7+
y6++
y2+
y7++
y8++
intensity
m/z
0
20
60
100
200
400
600
800
1000
YIEESAASKR+XL_L
B_b_ion
B_y_ion
y8+
y3+
y4+
prec
b2+
b3+
y2+
y8++
b4+
y6+
b6+
b7+
y7+
b9+
intensity
m/z
0
200
400
600
200
400
600
800
1000
